# Apparent basicities of the surfaces characterizing the dominant crystal habits of distinct polymorphic forms of 4-aminosulfonamide

**DOI:** 10.1007/s00894-014-2276-7

**Published:** 2014-06-17

**Authors:** Piotr Cysewski

**Affiliations:** Department of Physical Chemistry, Collegium Medicum of Bydgoszcz, Nicolaus Copernicus University in Toruń, Kurpińskiego 5, 85-950 Bydgoszcz, Poland

**Keywords:** Polymorph, Morphology, Sulfonamide drugs, Morphology growth, Chromogenic molecular probe, Thymol blue

## Abstract

A new approach for estimating local basicities/acidities of groups exposed on crystal surfaces was formulated and validated. The model, constructed within a quantitative structure–property relationship (QSPR) framework, allowed the expression of the protonic properties of amine and sulfonamide groups as functions of simple molecular descriptors of geometric types. This enabled the application of a QM/MM approach for the structural optimization of SNM molecules located on the surfaces of the dominant crystal habits. The obtained pKa values were used for classification of the protonic properties of four *p*-aminosulfonamide (SNM) polymorphs. The computed distributions of the surface p*K*_a_ values suggested that, for all polymorphs, the amino group has statistically the same proton-accepting ability on the crystal surface as in bulk water solution. Although sulfonamide groups on the crystal surface—especially those distributed on β- and γ-dominant faces—seem to be more acidic compared to bulk water solution, the p*K*_a_ values are statistically indistinguishable irrespective of the morphology. This suggests that experimentally observed differences in the perichromic properties of SNM polymorphs do not arise from local pH changes, Thus, apparent local basicities are to be relaed to structural similarity of SNM surfaces and thymol blue conformers anabling direct interactions.

**Electronic supplementary material:**

The online version of this article (doi:10.1007/s00894-014-2276-7) contains supplementary material, which is available to authorized users.

**Figure:**
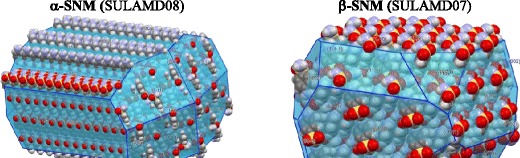
Experimentally observed differences in the perichromic properties of *p*-aminosulfonamide polymorphs are structural in origin; they are not due to local pH changes

## Introduction

The fact that active pharmaceutical ingredients (APIs) can adopt divers polymorphic forms has important therapeutic, scientific, commercial, and industrial significance [1]. The ability of many organic molecules to form crystals with different arrangements or molecular conformations affects variety of physical properties, including spectroscopic, thermodynamic, kinetic, mechanical, and surface characteristics [2]. Furthermore, the different polymorphs can very often have different bioactivities, defined in terms of dissolution rates and shelf-lives, due to alterations in chemical reactivity [3]. The preparation of new polymorphic forms also encourages innovation due to the ability to protect intellectual property by patenting the form of a crystal as well as its chemical formula and processing methods associated with it [4]. Among many interesting aspects of polymorphism, protonic properties are worth mentioning due to their practical implications [2, 5–7]. First of all, oral administration is the most commonly drug-delivery route. However, the variability of gastrointestinal pH has been shown to present a serious challenge to the predictable release and bioavailability of active pharmaceutical compounds [8]. Thus, pH modifiers are often introduced into pharmaceutical technology in order to enhance the release of weakly basic drugs from swellable tablets [9, 10]. Also, when solid dosage forms are used, the decomposition rates of many APIs can be affected by the pH of the local environment of the solid [8, 11]. Surface acidity has been proven to affect the chemical reactivities and physical stabilities of lyophilized solids [12]. This is why the role of amorphous solid forms has been highlighted [13, 14]. Recently [15], the importance of microenvironment acidity in the context of the variation in the protonic activity of *p*-aminosulfonamide (SNM) as a function of its polymorphic form and morphology was addressed. Although sulfonamide drugs have been somewhat superceded by antibiotics, they are still extensively used to treat certain infections caused by some fungi, protozoa, and both Gram-positive and Gram-negative microorganisms [16, 17]. Sulfonamide drugs that act as antibacterial and antimicrobial agents inhibit bacterial growth and activity due to their ability to interfere with metabolic processes in bacteria. They are used in the prevention and treatment of bacterial infections, diabetes mellitus, edema, hypertension, and gout [17]. Sulfonamide derivatives are remarkably polymorphic [18–36]. For example, four distinct polymorphs of SNM have been obtained: α (Pbca) [19–22], β (P21/c) [23–25], γ (P21/c) [20, 25, 26], and δ (Pbca) [27]. They have additional interesting features that have beenstudied experimentally by diffuse reflectance spectroscopy [37]. Sulfonamides, along with other drugs, can exhibit different perichromic properties depending on the polymorphic form adopted. This ability to selectively cause adsorbed dyes to change color can be used for the quantitative assessment of polymorph composition. For example [15], the spectroscopic properties of thymol blue (TB) differ markedly depending on whether it is adsorbed on β- or γ-SNM crystals. This anables to use this indicator as an effective molecular probe for analytical purposes. These remarkable differences in the degree of protonation of thymol blue suggest that γ-SNM is significantly more basic than the β-form (by >3 pH units) [15]. Two models were proposed to explain the observed phenomena. The first relies on the values of the dissociation constants of the indicator and sulfonamide. The water solution of SNM is slightly acidic (pH = 6.27 [38]), which is obviously related to the fact that the amino group linked to the sulfonic fragment gains the ability to donate a proton (p*K*_a_ = 9.4 [39]), with the aniline group remaining in its neutral form (p*K*_a_ = 2.1 [40, 41]). The crystal surface is rich in proton-accepting aniline groups and proton-donating sulfonamide fragments. Under normal humidity, the latter may be partly deprotonated in the presence of even a small number of water molecules. The mechanism by which SNM exhibits its basic behavior is expected to be related to interactions of these fragments with thymol blue. It is reasonable to expect that the sulfonic group of TB, which is quite an acidic group (p*K*_a_ = 1.7 in water [42, 43]), will be responsible for protonating the aniline group of SNM. On the other hand, the phenolic hydroxyl group (p*K*_a_ = 8.9 in water [42, 43]) is able to, at least partly, increase its proton donation to deprotonated sulfonamide fragments. This double proton transfer from TB to one or two SNM molecules leads to a dianionic form what is responsible for the observed changes in the absorption spectra and the blue tinge. This model was validated by the observation that the active centers of the thymol blue conformers and the basic/acidic sites on the SNM polymorph show the striking structural similarities. Thus, the differences among the SNM polymorphs in apparent surface pH can be explained in terms of the structural properties of the molecular probe and the adsorbing surface. Consequently, SNM polymorph recognition based on ion-pair formation with the molecular probe can be related to morphological differences between the adsorbing surfaces [15]. There is another way of explaining the observed apparent differences in the local pH values of β- and γ-SNM crystals, using a one-site model. Dissociation of the sulfonic group of thymol blue is prompted by the presence of trace adsorbed water, and one of phenolic groups interacts with the sulfonamide fragment of SNM. In this case, only differences between both polymorphs in the surface concentration of the sulfonamide fragment can lead to the different values of apparent pH for . In either model, it is assumed that the protonic properties (p*K*_a_ values) of the active groups (−NH_2_ or –SO_2_NH_2_) are not affected by the crystal packing and exposure on the surfaces of different crystal habits. If these factors significantly influence the dissociation constants, the formation of ion pairs through direct interactions between indicator and surface may be prohibited. Thus, the aim of the work was to quantify the influence of the intermolecular interactions that occur in the crystals formed by all known SNM polymorphs on the proton-donating and proton-accepting abilities of sites located on the surfaces of the most morphologically important crystal habits.

## Computational methods

A two-step procedure was applied to characterize the morphology- and polymorphism-related variations in the protonic properties of the *p*-aminosulfonamide polymorphs (SNM). In the first stage, a quantitative structure–property relationship (QSPR) approach was used to formulate a linear model that employed geometry-based molecular descriptors. The Cambridge Structural Database (CSD) [44] provides data deposited in CIF files. The contents of these files include molecular geometry, spatial arrangements, and information about crystal symmetry (such as the size and shape of the unit cell augmented by a list of symmetry operators). This set of data unambiguously defines not only the crystal lattice of any polymorphic form but also morphology-related habits. This is why the QSPR model defined here was based exclusively on geometric parameters. On the other hand, the data deposited in the Reaxys database [45] was used to formulate two training sets of molecules. The present work used the available p*K*_a_ values that characterize the proton-donating properties of all the analyzed species. It is worth mentioning that the p*K*_a_ values provided for the aniline group highlight its readiness to undergo anilinium cation deprotonation and form a neutral amine group, while those for the sulfonamide group indicate the tendency of the amine site to deprotonate, leading to an anionic form of SNM or its analogs. After validating the empirical model, a second step involved the application of the QM/MM approach to assess the protonic properties of acidic/basic centers located on the most probable morphology-related crystal habits of each of the four polymorphic forms of SNM.

### QSPR model construction

Reaxys database was searched for available p*K*_a_ values of aromatic amines and sulfonamide acids measured in water solution at room temperature. Eighteen benzene sulfonic acid analogs and 51 aromatic amines were found. Only *meta*- and *para*-substituted analogs were used to mimic properties of the *p*-aminosulfonamide. Analogs with substituents at *ortho* position in the aromatic ring can sterically interfere with amino and sulfonamide groups, directly affecting their geometric properties. The QSPR approach relies on relationships between molecular descriptors and the p*K*_a_ values of the amine and sulfonamide groups connected to the aromatic ring. In cases where several values had been reported by different authors, the mean p*K*_a_ values were used. Detailed information about the data collected is provided in the “Electronic supplementary material” (ESM; see Tables S1 and S2 for benzene sulfonic acid and aniline analogs, respectively). The geometries of all of the molecules considered were fully optimized at the B3LYP/6-311+G** level with the aid of the G09 program [46]. The “ultrafine” option was used for grid density, along with the “very tight” option for optimization. When thermodynamic computations were performed, checks were carried out to ensure that no imaginary frequencies were assigned and that the structures obtained represented ground states. The solvation model was applied within the PCM framework [47], which included explicit hydrogen centers with Bondi parameterization [48]. In this approximation, the molecule is considered to be present inside a cavity, and the solvent is represented as a structureless continuum. Such a model has proven to be sufficiently accurate to provide successful p*K*_a_ predictions [49–53]. After optimization, a variety of molecular descriptors were computed and tested within the QSPR approach. Among the parameters considered were those derived directly from geometric properties such as bond lengths, bond angles, and torsion, geometry-related measures of π-electron delocalization in the substituted aromatic ring, and other characteristics obtained from single-point computations performed at the same level of theory (such as Mulliken charges on side-group atoms, polarizabilities, frontier orbital energies, dipole moments, ionization potentials, and chemical hardnesses and affinities). The resulting set of descriptors was subjected to a statistical analysis performed in Statistica [54, 55] to check the reliability of the obtained models. This particular software package was selected for use as it enabled direct assessments of not only the model’s accuracy but also the statistical significance of coefficients in linear regressions. The best models obtained in this manner were used to predict the p*K*_a_ values of morphology- and polymorphism-related SNM properties.

### Surface basicity/acidity assessments

**Fig. 1:**
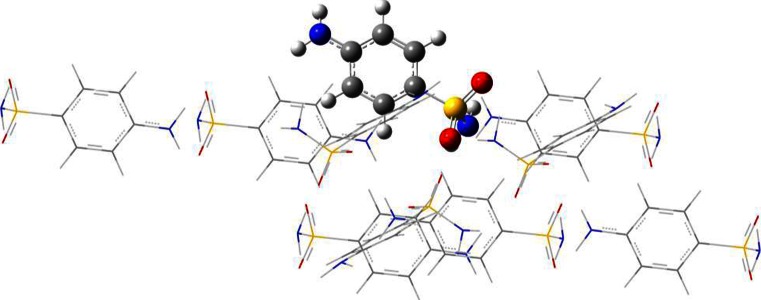
Schematic representation of the QM/MM model used to predict the structural properties of individual SNM molecules located on the surfaces characterizing the most dominant faces. Here, a (110) surface slip at a fractional coordinate of 0.471 is shown for the β-SNM form (SULAMD07)

The crystal habits for all four SNM polymorphic forms were estimated using the growth morphology method [56, 57], as implemented in Accelrys Material Studio 6.1 [58]. Before the actual morphology modeling was performed, the crystal geometries were optimized using the DMol3 module with delocalized internal coordinates for periodic systems [59]. Full gradient optimization was performed, including the molecular geometry, while the cell parameters were not optimized (the experimental values reported in the CIF files were kept constant). The Perdew–Burke–Ernzerhof correlation functional (PBE) [60] combined with the double numerical basis set was used, with *d*-function polarization on all heavy atoms and the *p*-polarization function on all hydrogen atoms. This is important for obtaining a proper description of hydrogen bonding. Additionally, due to the presence of electron delocalization and potential stacking, corrections for dispersion interactions were included using Grimme protocol [61, 62]. This approach to crystal optimization is a satisfactory compromise between accuracy and computational cost [63]. No significant geometric changes were observed after optimization, suggesting that the structural parameters provided by the CIF files were accurate and reliable. The results of crystal optimization are supplied in the ESM (see section S.III). The structures obtained were used to generate the molecular coordinates of each of face identified by morphological modeling [64]. Among many theoretical approaches that permit the prediction of bulk crystal shape from atomic structure, the growth morphology method [64, 65] was selected for the present work. This method assumes that the growth rate of a crystal face is proportional to its attachment energy (*E*_att_), defined as the energy released on the addition of a slice to a growing crystal surface. In this model, the morphologically important faces are those with the lowest attachment energies, since they show the slowest growth rate (the lower the *E*_att_ value, the higher the probability of slipping along such face). The attachment energy is calculated for a series of slices defined by their (*hkl*) coordinates, using the “ultrafine” option for energy and face estimation. After identifying the dominant faces, each SNM molecule on each surface was optimized using the QM/MM approach. One of the SNM molecules exposed on the surface of a particular habit was defined as being in the first region (typically denoted the “high part”). Then, all of the molecules within 10 Å of the first one were included in the second region (termed the “low part”). A schematic representation of this model is provided in Fig. 1.

The three-parameter Lee–Yang–Parr exchange-correlation functional, B3LYP, was used for geometry optimization. Different basis sets were used for the high and low parts as follows: ONIOM B3LYP/6-311 + g (d,p)//B3LYP/6-31 g (d,p)), where “ONIOM” is the QM/MM implementation in G09 and the subsequent acronyms denote the levels of theory used for high and low part computations, respectively. Two additional simplifications were used. In order to use the same method of SNM geometry optimization as employed for molecular descriptors in the QSPR model, the polar solution was mimicked using the PCM framework with Bondi parameters. Thus, the whole cluster was “immersed” in the electrostatic field of implicit water molecules. This is obviously a serious simplification due to the omission of any explicit water molecules that could interact and affect the geometry of the SNM molecule. Of course, the same shortcoming arises in bulk solvent modeling if the PCM framework is adopted. However, it is necessary to reduce the problem in this manner in order to make computations feasible. It is reasonable to expect that the same error is introduced in both cases (in bulk solution and with the crystal surface exposed to bulk solution). Additionally, surface rigidity was assumed. Thus, only partial optimization of the analyzed clusters was performed, involving full minimization of only one SNM molecule surrounded by its nearest-neighboring molecules. It is worth mentioning that, even after implementing these assumptions, the geometry optimizations were quite time consuming due to the rather slow convergence of the gradient minimizations. Different incongruent effects arising from interactions with explicit neighbors and the implicit environment complicate the problem of minimization as they lead to many shallow minima. In total, 87 optimizations were performed, providing a reasonably representative probe of the SNM molecular geometries on the surfaces of different crystal habits. The number of optimizations performed depended on not only the number of faces identified and their multiplicities but also the number of unique monomer orientations on each surface.

## Results and discussion

*p*-Aminosulfonamide is a molecule with proton-accepting and proton-donating properties due to the presence of amino and sulfonamide groups. These fragments can form ion pairs with other molecules if the conditions required by the corresponding p*K*_a_ values are met. For example, thymol blue, which is used as a chromogenic probe of SNM surfaces [15, 38], exhibits different affinities toward β and γ polymorphs based on how blue-tinged the surface is. It is known that this is associated with the deprotonation of both sulfonic groups and one phenolic group of TB. Both of the suggested mechanisms [15] involved direct interactions with SNM in these proton-exchange reactions. However, it is not obvious that amino and sulfonamide groups have the same properties when different intermolecular interactions are present on different surfaces (as defined by the type of polymorph and the morphology). Thus, the main motivation for this work was to quantify the diversity of p*K*_a_ values due to local structural heterogeneities. There are many approaches that can be employed to theoretically predict the acidity constants of chemical substances, from ab initio or first-principles quantum chemistry protocols through to semiempirical computations and empirical parameterization schemes. The most commonly applied approach relies on a thermodynamic cycle [49, 50] involving proton exchange reactions in the gas phase and in solution. This approach has the advantage of being able to compensate for two main sources of errors: those arising from gas-phase proton affinity inaccuracies and those in the computed solvation enthalpies of protonated and deprotonated species as well as the proton itself. This methodology has been successfully applied [49–53] to a variety of cases, especially those in which a high level of computation is combined with appropriate parameterizations of solvent–solute interactions. Unfortunately, it was not possible to use such scheme in this project due to the ambiguity in the definition of the thermodynamic cycle in the case of surface-exposed molecules. Thus, another strategy was adopted here. In the first step, empirical formulas relating p*K*_a_ values to some geometric and electronic features of SNM were defined via the QSPR protocol. After validating the resulting linear regressions, these functional relationships were used to analyze the protonic features of SNM on surfaces.

## Geometry-based model for predicting p*K*_a_ values of sulfonamide analogs

**Fig. 2:**
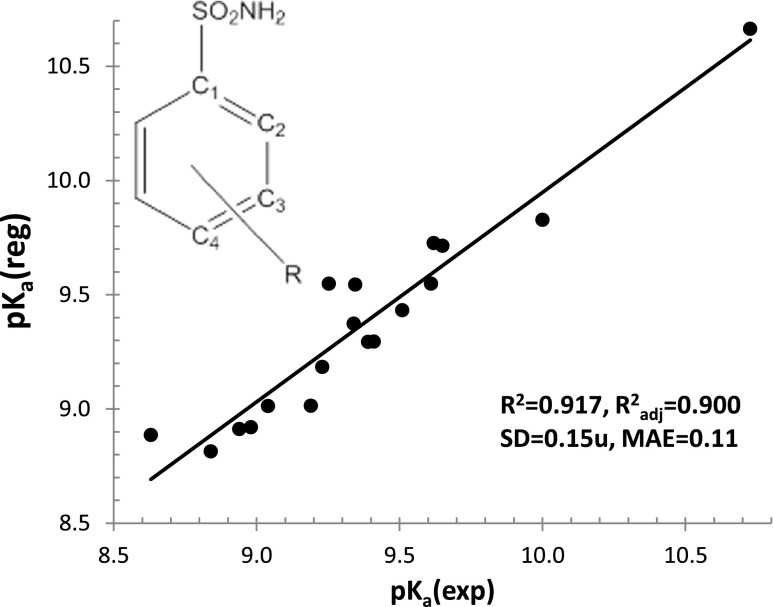
Graphical illustration of the QSPR model used to predict p*K*_a_ values for series of sulfonamide derivatives in water solution (at 25 °C). *R* ^2^_adj_ is the square of the correlation coefficient adjusted for the number of data points, and “u” is a p*K*_a_ unit

The training set contained 18 *meta*- and *para*-substituted analogs of benzenesulfonamide available in the Reaxys database. The characteristics of these compounds are presented in the ESM (Table S1). Among the many variables used for linear regression analysis, three parameters seem to be quite effective at predicting p*K*_a_ values. According to the formula provided in Fig. 2, it is possible to relate dissociation constants to the length of the S–N bond within the sulfonamide group, the degree of π-electron delocalization expressed in terms of the Dewar-type resonance energy (DRE), and the chemical hardness (denoting the difference in the energies of the frontier orbitals). Interestingly, the influences of these molecular descriptors were also proven to be statistically significant factors contributing to aromaticities of substituted 5-, 6-, and 7-member rings [65] in polar media. As presented in Fig. 2, the value of the correlation coefficient is quite acceptable, *R* ^2^ = 0.917. Other characteristics of model validation were also encouraging. For example, the standard deviation was about 0.15 pH units and the mean average error (MAE) was as low as 0.11 pH units. Thus, it seems that, within this level of precision, the alteration of the geometry of the sulfonamide group attached to the aromatic ring can be related to changes in proton-donating properties.

1 $$ \mathrm{p}{K}_{\mathrm{a}}(i)=105.8\cdot {d}_{\mathrm{S}-\mathrm{N}}(i)+1.374\cdot \mathrm{DRE}(i)-0.00754\cdot \eta -183.0. $$

## Model of aromatic amine group protonic properties

**Fig. 3:**
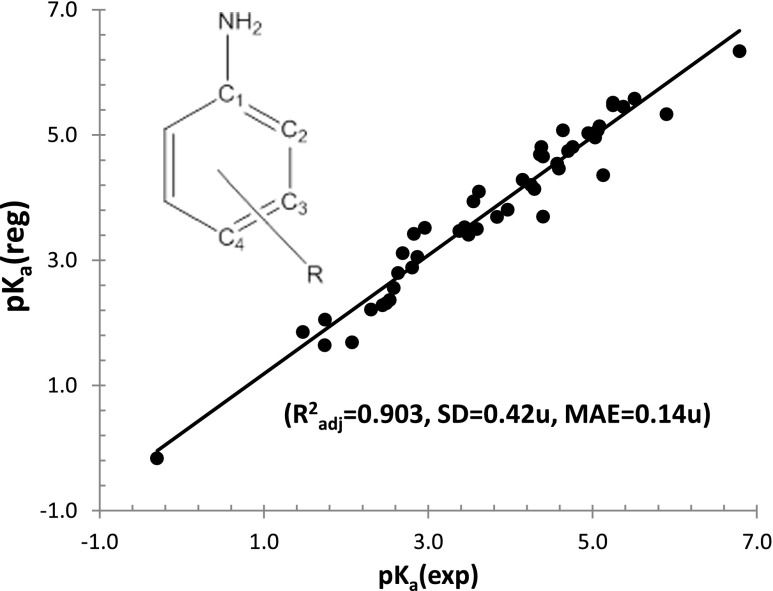
Graphical illustration of the QSPR model used to predict p*K*_a_ values for set of 51 aniline derivatives in water solution (at 25 °C). Notation is the same as used in Fig. 1

Encouraging results in relating the p*K*_a_ value of the SO_2_NH_2_ group to its geometry and frontier orbitals justified an attempt to characterize the aromatic amine group in a similar manner. In this case, the Reaxys database contained far more compounds that could be used in training set construction. Again, restricting the species to *meta*- and *para*-substituted anilines measured in water solution at room temperature resulted in the selection of 51 compounds. The set was quite heterogeneous and comprised aniline derivatives with quite diverse proton-accepting properties. The ESM (see Table S2) lists the relevant experimental and predicted *p* *K*_a_ values as well as parameters used in QSPR model. As shown in Fig. 3, a linear regression model was constructed based on the length of the C–N bond (amine group attached to the aromatic ring) and two methods of expressing π-electron delocalization in terms of resonance energy: the Dewar (DRE) and the total (TRE) resonance energy. These parameters are defined in the ESM (see section S.II). The correlation coefficient (*R* ^2^ = 0.909) was found to be of the same quality as previously obtained for the sulfonamide group. The mean average error value was also acceptable (MAE = 0.14). This time, however, the standard deviation was slightly worse: 0.42 pH units. This defines the limit on the precision of the proposed model relating p*K*_a_ values to variations in the molecular descriptors of aniline derivatives.

## Morphologies of SNM crystal polymorphs

**Fig. 4:**
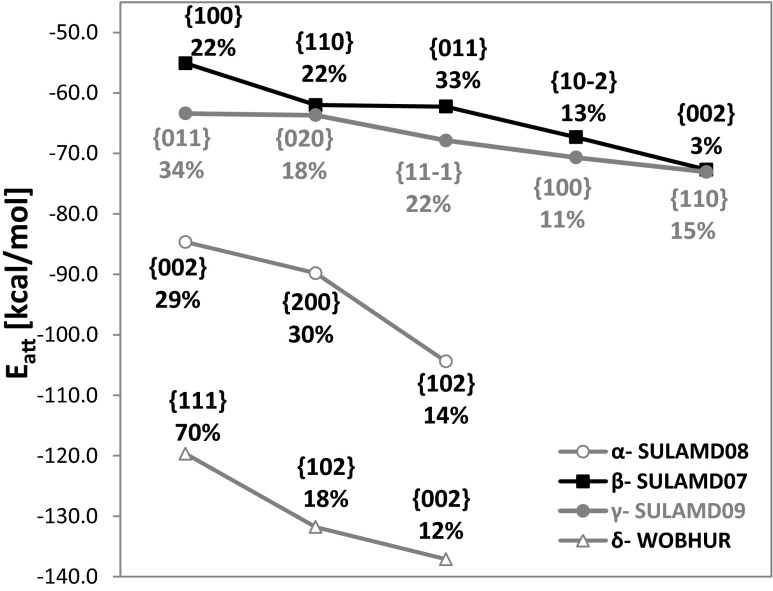
Attachment energies and percentages of the surface area for the most morphologically important faces of all four SNM polymorphs

**Fig. 5:**
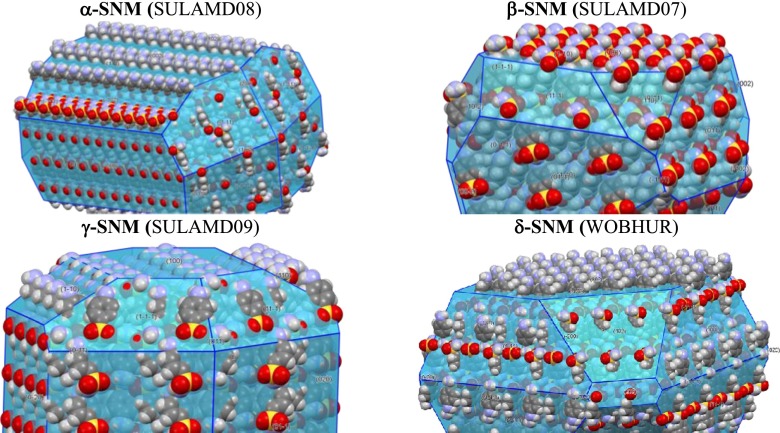
Schematic representation of different arrangements of SNM molecules on surfaces characterizing the dominant habits of different polymorphs

As mentioned before, sulfonamide drugs exhibit remarkably high polymorphism. Eleven records of SNM were found in the CSD. Although all of these species can be categorized using only two space groups, unique molecular arrangements led to the classification of the SNM crystals into four distinct polymorphic forms. As shown in Table 1, some similarities can be expected between two pairs of polymorphs irrespective of the temperature at which the X-ray diffraction measurements were obtained. Indeed, the cell volume is twice as large for α- and δ-SNM than for β- and γ-SNM. The CSD reference codes shown in bold in the first column of Table 1 indicate that the corresponding structures were obtained with the highest accuracy, probably due to the low temperature at which the measurements were taken. These structures were used for pre-optimization and detailed morphology analysis. In order to determine the local protonic properties of SNM, it is necessary to know how the *p*-aminosulfonamide molecules are arranged on the actual surface. In this context, the dominant faces are believed to dictate the physicochemical properties of crystals. Based on the growth morphology method applied here, information was obtained for the dominant faces of all four SNM polymorphs. Similarities in cell volume are also resemble similarities in the attachment energy. The β and γ forms are characterized by very similar values of *E*_att_. On the contrary, the attachment energies of α- and especially δ-SNM are much lower, suggesting higher resistances to slipping and consequently higher overall hardness. A list of the dominant faces is provided in Fig. 4, along with predicted percentages of the total area of each crystal habit. It is not surprising that crystals of different polymorphic forms can also differ in their dominant habits, This, in turn, can lead to significant structural changes in the surfaces exposed to the solution, as presented schematically in Fig. 5. Even from this qualitative description, it is clear that the exposure of proton-donating and proton-accepting groups to the solution can be significantly influenced by both crystal morphology and polymorphism. In order to quantify this effect, the advantages of the QM/MM approach were utilized.

**Table 1:** Experimental parameters defining the structural properties of *p*-aminosulfonamide crystals. Structures used for QM/MM computations are highlighted in boldface

CCSD code	Polymorph	*V*	*a*	*b*	*c*	α = γ	β	r***	*T* (K)
**SULAMD08** [21, 22]	α*	1503.6	14.60	5.57	18.48	90.0	90.0	0.034	150
SULAMD05 [23]	α*	1537.8	5.62	18.50	14.79	90.0	90.0	0.135	298
SULAMD [24]	α*	1547.1	5.65	18.51	14.79	90.0	90.0	0.135	298
**SULAMD07** [25]	β^**^	738.3	8.87	8.92	9.96	90.0	110.4	0.032	150
SULAMD03 [23]	β^**^	755.3	8.98	9.01	10.04	90.0	111.4	0.049	298
SULAMD04 [24]	β^**^	755.3	8.98	9.01	10.04	90.0	111.4	0.080	298
SULAMD06 [26]	β^**^	753.5	8.97	9.00	10.04	90.0	111.5	0.062	298
SULAMD01 [27]	β^**^	758.7	9.00	9.02	10.05	90.0	111.5	0.110	298
**SULAMD09** [22]	γ^**^	748.2	7.82	12.99	7.62	90.0	104.9	0.036	150
SULAMD02 [28, 29]	γ^**^	768.7	7.95	12.95	7.79	90.0	106.5	0.092	298
**WOBHUR** [30]	δ^*^	1507.0	9.71	8.68	17.89	90.0	90.0	0.042	150

^*^Space group 14 (“P 21/c”), ^**^ space group 61 (“P bca”); ^***^the r-factor indicates the accuracy of the refinement protocol

2 $$ \mathrm{p}{K}_{\mathrm{a}}(i)=137.3\cdot {d}_{\mathrm{C}-\mathrm{N}}(i)+2.244\cdot \mathrm{DRE}(i)-0.339\cdot \mathrm{TRE}(i)-196.3. $$

## Diversity of polymorphism and morphology-related local basicities

**Fig. 6:**
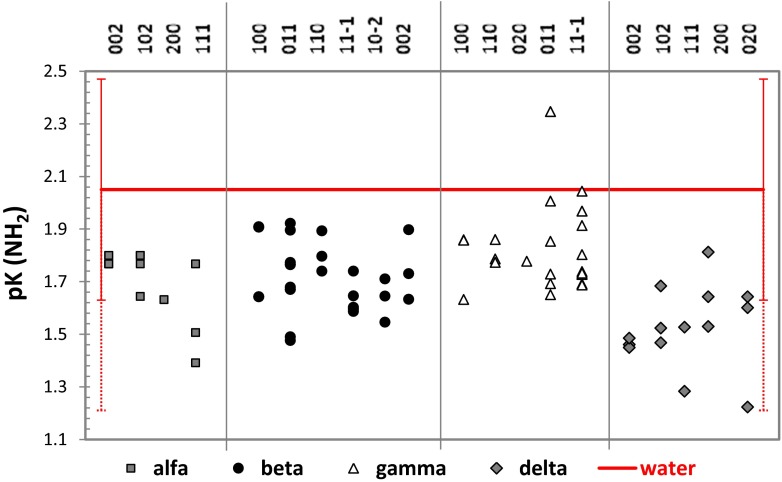
Diversity of the p*K*_a_ value of the aromatic amino group present in the dominant faces of all four SNM polymorphs

**Fig. 7:**
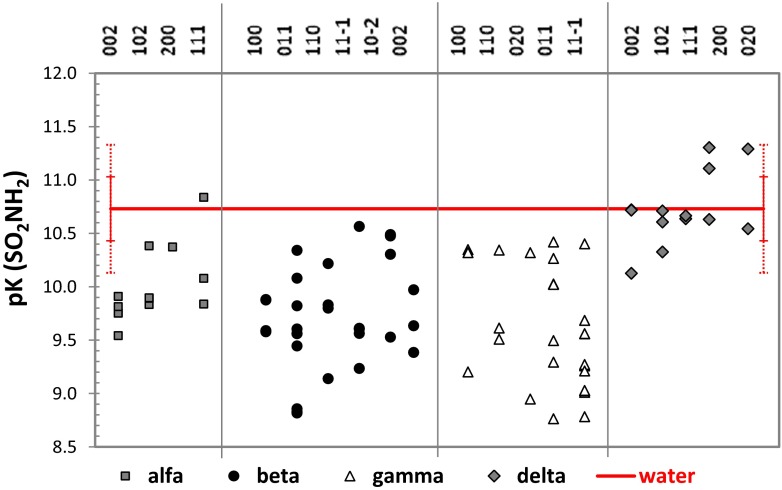
Diversity of the p*K*_a_ values of the sulfonamide group present in the dominant faces of all four SNM polymorphs

According to the QSPR model, it is possible to estimate the protonic properties of sulfonamide or amine groups attached to the aromatic ring using only geometric parameters. This fortunate circumstance enables us to use QM/MM computations for direct evaluation of e the structural diversity of SNM molecules contituting morphologically dominant faces. Figures 6 and 7 present the results of of such analysis. The experimental values of p*K*_a_ measured in water for both the amino and sulfonamide groups are represented by bold lines. The standard deviations that characterize the precision of the QSPR model are also provided. Inspecting Figs. 6 and 7 leads to quite interesting conclusions. The aromatic amino group (a weak base) is deprotonated in water solution (p*K*_a_ = 2.1 [40, 41]). Similar values are expected on the surfaces, regardless of the crystal habit and polymorph considered. However, a systematic shift toward slightly lower p*K*_a_ values is apparent. This seems to be statistically significant since most of the values obtained are below the SD threshold. However, if the precision is increased by extending the tolerance to double the standard deviation (which means increasing the confidence interval from 68.3 % to 95.5 %), it becomes clear that intermolecular interactions on the crystal surface do not significantly alter the affinity of the aniline group of SNM for protons. Interestingly, this conclusion holds for all of the habits considered for each analyzed polymorphic form. Thus, the ability of the amine group to form an ion pair with thymol blue (the molecular probe) is approximately the same as it is in bulk solution. We can expect that the sulfonic group of TB (p*K*_a_ = 1.7 [42, 43]) can protonate amine groups on the surface, but phenolic groups (p*K*_a_ = 8.9 [42, 43]) have no such ability. Furthermore, the heterogeneity of the p*K*_a_ values of the sulfonamide substituent is shown in Fig. 7. This group is weakly acidic in water solution (pH = 6.27 [38]) and remains so on all of the surfaces considered here. The δ polymorph is the only one for which no significant alteration in sulfonamide acidity is observed when compared to its acidity in water solution. For the other polymorphs, it is possible to find a population of structures characterized by statistically significantly stronger acidities compared to water solution. This suggests that there are some faces with acidities are significantly different to those in bulk water solution. In this context, the noticed few outliers (by at least two standard deviations) suggest changes in the acidities of the SNM surfaces. This non-negligible effect is as strong as two pH units. Interestingly, the most pronounced increases in SNM acidity are observed for the β and γ polymorphs. Not only do these polymorphs present stronger reductions in the p*K*_a_ values, but all of their morphologically important habits show this property. This suggests that the proton-donating ability of sulfonamide is strengthened by the crystal surface, enhancing the formation of SNM–TB pairs. This is an important conclusion supporting previously proposed mechanism [15] explaining the apparent basicities of sulfonamide crystals. Thus, the SNM surfaces show proton-accepting behavior towards thymol blue, leading to the dianionic form of this indicator. Since we do not expect any significant changes in the p*K*_a_ values of the most dominant faces of the β and γ polymorphs, the distribution of active groups on the surface is a crucial factor. The distribution of amino and sulfonamide groups on the γ polymorph is more similar to that of the sulfonic and phenolic groups of the TB conformers. Thus, recognition through ion-pair formation is more pronounced for this polymorph than for β-SNM.

## Conclusions

The observed Intriguing differences in the apparent basicities of SNM polymorphs [15, 38] initiated this project, in which the variations in the p*K*_a_ values characterizing local protonic equilibria were estimated. As previously suggested [15], the interactions of thymol blue (serving as a chromogenic molecular probe) were significantly influenced [38] by the polymorphic form. This resulted in changes of the color of the crystal powder after dyeing. Unfortunately, the actual mechanism by which thymol blue binds to the sulfonamide surface is unknown. Modeling of the direct interactions of this chromosphere with SNM molecules on the crystal surface is not a trivial task, since the number of possible structures to consider rules out a simple and quick solution. It is necessary to take into account the three species of thymol blue with different degrees of deprotonation as well as their flexibilities and their structural differences. Besides, there are several stable faces for each sulfonamide polymorph, In this context, information inferred from the relative p*K*_a_ values of thymol blue and sulfonamide seems to be a rational first step simplification explaining the differences in the chromogenic characteristics of the SNM polymorphs. The QSPR model proposed here allowed to quantify the influences of neighboring molecules on the proton donation and acceptance of *p*-aminosulfonamide. To the author’s best knowledge, such a detailed analysis was not performed to date. Besides, the methodology proposed in this paper may prove useful for more general purposes. Obviously, the conclusions drawn from the computations presented here are as precise as the initial model that related the crystal morphology and polymorphism to the dissociation constants. Developing other, more general, models seems to be a promising subject for further exploration. The most important conclusion drawn from the work is that surface perichromism is a strictly recognition-based phenomenon rather than an acid–base one. This means that the surface may be recognized as basic by some chromogenic probes and as neutral by other ones. The computed distribution of the surface p*K*_a_ values supports this notion by demonstrating that the amino group has statistically the same proton-accepting ability as it does in water solution. Although sulfonamide groups—especially those distributed on the dominant faces of the β and γ polymorphs—appear to be more acidic on the crystal face than in water solution, but this change does not distinguish both polymorphs. Thus, aciditiy change is not the reason for the observed differences in the perichromic properties of these two crystals after dyeing by thymol blue. As it was explained in the previous study [15], structural differences are expected to be the source of the observed differences in surface recognition by thymol blue, Furthermore, slips along different faces are most likely to occur in β- and γ-SNM, but their attachment energies are very similar. Predicted values of *E*_att_ suggest the following order of hardness for SNM polymorphs: β ∼ γ,> α >> δ.

## Electronic supplementary material

Below is the link to the electronic supplementary material.

ESM 1 (DOC 183 kb)
