# Supplementary material for: Apparent basicities of the surfaces characterizing the dominant crystal habits of distinct polymorphic forms of 4-aminosulfonamide
Source: J Mol Model. 2014 Jun 17;20(7):2276. doi: 10.1007/s00894-014-2276-7 (PMC4107284; doi:10.1007/s00894-014-2276-7)
Supplement: Supplementary file 1 — (DOC 183 kb) [file 894_2014_2276_MOESM1_ESM.doc]

**Supporting materials**

**I. Definition of molecular descriptors selected for QSPR model**

***Electrophilicity Index***

The density functional theory provides rigorous definitions of intuitive chemical concepts such as electronegativity (χ) and hardness (η) [1]. For an N-electron system with total energy E and external potential V(r) the former quantity can be defined as follows [2]

$$\chi=-\left(\frac{\partial E}{\partial N}\right)_{V(r)}$$

Hardness (η) is defined [3] as the corresponding second derivative,

$$\eta=\left(\frac{\partial^{2}E}{\partial N^{2}}\right)_{V(r)}$$

It is interesting to note that in the case of assuming the validity of Koopmans' theorem [4] electronegativity and hardness can be directly related to frontier orbital energies,

$\chi=-\frac{\varepsilon_{HOMO}-\varepsilon_{LUMO}}{2}$ and $\eta=\varepsilon_{HOMO}-\varepsilon_{LUMO}$

where $\varepsilon_{HOMO},\varepsilon_{LUMO}$denote highest occupied and lowest unoccupied molecular orbitals, respectively. This makes the results of single point energy computations as direct source of values of these molecular descriptors.

# 1. Chattaraj PK, Sarkar U, Roy DR (2006) Electrophilicity Index. Chem Rev 106: 2065–2091

# 2. Parr RG, Donnelly RA, Levy M, Palke WEJ (1978) Electronegativity: The density functional viewpoint. Chem Phys 68:3801-3807

# 3. Parr RG, Pearson RGJ (1983) Absolute hardness: companion parameter to absolute electronegativity. Am Chem Soc 105:7512-1516

4. Koopmans TA (1933) Ordering of Wave Functions and Eigenenergies to the Individual Electrons of an Atom. Physica 1:104-113

***Energetic criterions of aromaticity***

The Dewar type resonance energy (DRE) or total resonance energy (TRE) are forms of expressing amount of π-electron delocalization based on concept of resonance energy [1, 2]. Since the bond energy is bond length dependent, the harmonic oscillator stabilization energy (HOSE) is defined as a negative value of the energy necessary to deform the geometry of the real molecule into the geometry of one of its Kekulé (or resonance) structures with localized single and double bonds [3]. The energy of deformation has been derived from a simple harmonic oscillator potential

$$E_{def}(i)=-\frac{1}{2}\cdot\left[\sum_{r=1}^{n1} \left(d_{r}^{'}-d_{0}^{S}\right)\cdot k_{r}^{'}+\sum_{r=1}^{n2} \left(d_{r}^{"}-d_{0}^{d}\right)\cdot k_{r}^{"}\right]$$

where *d_r_’* and *d_r_”* stand for the lengths of π bonds in the real molecule, whereas n_1_ and n_2_ are the numbers of the corresponding formal single and double bonds in the *i*th canonical structure, respectively. In the process of deformation, the n_1_ bonds corresponding to the single bonds in the *i*th canonical structure are lengthened whereas the n_2_ bonds corresponding to the double bonds in the *i*th canonical structure are shortened to the reference bond lengths *d_o_^S^* and *d_o_^d^*, respectively. The interesting feature of HOSE model is the possibility of estimation of *i*th canonical structure contributions to aromatic stabilization energy, HOSE*_i_* [3,4] as well as the total stabilization energy, after applying the following equation:

$$1/HOSE=\sum_{i=1}^{n} 1/HOSE_{i}$$

The resulting values correspond to the Dewar type resonance energy (DRE) or total resonance energy (TRE) when using conjugated or isolated single and double bonds as a reference, respectively [5].

1. Pauling L, Wheland GW (1933) The Nature of the Chemical Bond. V. The Quantum‐Mechanical Calculation of the Resonance Energy of Benzene and Naphthalene and the Hydrocarbon Free Radicals. Chem Phys 1:362-375

2. Kistiakovski GB, Ruhoff JR, Smith HA, Vaughan WE (1936) Heats of Organic Reactions. IV. Hydrogenation of Some Dienes and of Benzene. J Am Chem Soc 58:146-153

3. Krygowski TM, Anulewicz R, Kruszewski J (1983) Crystallographic studies and physicochemical properties of π-electron compounds. III. Stabilization energy and the Kekulé structure contributions derived from experimental bond lengths. Acta Crystallogr B39:732-739

4. Ciesielski A, Krygowski TM, Cyranski MK, Balaban A (2011) Defining rules of aromaticity: a unified approach to the Hückel, Clar and Randić concepts. Phys Chem Chem Phys 13:3737-3747

5. Kotelevskii SI, Prezhdo OV (2001) Aromaticity indices revisited: refinement and application to certain five-membered ring heterocycles. Tetrahedron 57:5715-5729

**SII. Values of used for QSPR model validation**

**Table S1.** Collection of experimental and predicted values of pK_a_ characterizing 18 analogues of benzenesulfonamide in water solution (at 25°C). The values of molecular descriptors used in regression analysis are also provided.

|  | |  | **R** | **pK_a_(exp)** | **pK_a_(reg)** | **d_(S-N)_**  **[**Å] | **DRE** | **η** | | --- | --- | --- | --- | --- | --- | --- | | 1 | - | 9.41 | 9.29 | 1.671 | 12.11 | 141.4 | | 2 | 3-Me | 9.61 | 9.55 | 1.673 | 12.14 | 136.4 | | 3 | 4-Me | 9.51 | 9.43 | 1.672 | 12.11 | 134.1 | | 4 | 3,4-Me | 10.73 | 10.66 | 1.678 | 12.43 | 119.4 | | 5 | 4-NH_2_ | 9.34 | 9.37 | 1.670 | 12.26 | 140.5 | | 6 | 4-F | 8.84 | 8.81 | 1.665 | 12.18 | 131.4 | | 7 | 3-CN | 9.62 | 9.73 | 1.673 | 12.20 | 133.4 | | 8 | 3-OMe | 9.65 | 9.71 | 1.674 | 12.10 | 128.9 | | 9 | 4-OMe | 9.39 | 9.29 | 1.671 | 11.99 | 121.7 | | 10 | 3-Cl | 9.23 | 9.18 | 1.669 | 12.15 | 133.5 | | 11 | 4-Cl | 9.04 | 9.01 | 1.667 | 12.17 | 132.4 | | 12 | 4-NO_2_ | 8.63 | 8.89 | 1.664 | 12.20 | 107.7 | | 13 | 3-CF_3_ | 8.98 | 8.92 | 1.667 | 12.15 | 140.3 | | 14 | 3,4-Cl | 8.94 | 8.91 | 1.666 | 12.19 | 130.1 | | 15 | 4-PO(OH)_2_ | 10.00 | 9.83 | 1.674 | 12.12 | 108.0 | | 16 | 3-NO2 | 9.19 | 9.01 | 1.665 | 12.20 | 108.6 | | 17 | 3-MeNH2 | 9.35 | 9.54 | 1.673 | 12.08 | 129.0 | | 18 | 4-MeNH2 | 9.25 | 9.55 | 1.672 | 12.14 | 127.2 | |
| --- | --- | --- | --- | --- | --- | --- | --- | --- | --- | --- | --- | --- | --- | --- | --- | --- | --- | --- | --- | --- | --- | --- | --- | --- | --- | --- | --- | --- | --- | --- | --- | --- | --- | --- | --- | --- | --- | --- | --- | --- | --- | --- | --- | --- | --- | --- | --- | --- | --- | --- | --- | --- | --- | --- | --- | --- | --- | --- | --- | --- | --- | --- | --- | --- | --- | --- | --- | --- | --- | --- | --- | --- | --- | --- | --- | --- | --- | --- | --- | --- | --- | --- | --- | --- | --- | --- | --- | --- | --- | --- | --- | --- | --- | --- | --- | --- | --- | --- | --- | --- | --- | --- | --- | --- | --- | --- | --- | --- | --- | --- | --- | --- | --- | --- | --- | --- | --- | --- | --- | --- | --- | --- | --- | --- | --- | --- | --- | --- | --- | --- | --- | --- | --- | --- |

**TableS2.** Collection of experimental and predicted values of pK_a_ characterizing 45 meta- and para- analogues of aniline in water solution. The sum of length of N-H bond on amino group augmented by Julg (Aj) and Bird (I6) aromaticity indices were used as the most adequate molecular descriptors defining QSPR model.

|  | |  | **R** | **pK_a_(exp)** | **pK_a_(reg)** | **C-N** | **DRE** | **TRE** | | --- | --- | --- | --- | --- | --- | --- | | 1 | - | 4.59 | 4.46 | 1.399 | 12.17 | 54.76 | | 2 | 3-Me | 4.70 | 4.74 | 1.400 | 12.16 | 54.39 | | 3 | 4-Me | 5.09 | 5.14 | 1.403 | 12.14 | 54.48 | | 4 | 3-NH2 | 5.03 | 4.96 | 1.400 | 12.20 | 54.11 | | 5 | 4-NH2 | 6.79 | 6.34 | 1.413 | 12.13 | 54.53 | | 6 | 3-OH | 4.29 | 4.14 | 1.397 | 12.17 | 54.88 | | 7 | 4-OH | 5.51 | 5.58 | 1.409 | 12.13 | 55.21 | | 8 | 3-F | 3.38 | 3.46 | 1.392 | 12.39 | 56.35 | | 9 | 4-F | 4.57 | 4.54 | 1.401 | 12.34 | 56.54 | | 10 | 3-CN | 2.83 | 3.42 | 1.390 | 12.20 | 54.43 | | 11 | 4-CN | 1.75 | 2.05 | 1.373 | 12.68 | 54.76 | | 12 | 4-CHO | 1.74 | 1.64 | 1.367 | 12.87 | 54.75 | | 13 | 3-Et | 4.76 | 4.81 | 1.401 | 12.16 | 54.37 | | 14 | 4-Et | 5.07 | 5.07 | 1.403 | 12.15 | 54.46 | | 15 | 3-OMe | 4.25 | 4.20 | 1.398 | 12.05 | 54.46 | | 16 | 4-OMe | 5.38 | 5.45 | 1.408 | 12.02 | 54.78 | | 17 | 3-Cl | 3.59 | 3.50 | 1.392 | 12.26 | 55.62 | | 18 | 4-Cl | 3.97 | 3.80 | 1.395 | 12.22 | 55.69 | | 19 | 3-NO2 | 2.64 | 2.80 | 1.388 | 12.21 | 55.52 | | 20 | 3-OEt | 4.15 | 4.28 | 1.398 | 12.08 | 54.46 | | 21 | 4-OEt | 5.25 | 5.51 | 1.409 | 12.03 | 54.73 | | 22 | 3-COMe | 3.62 | 4.09 | 1.396 | 12.16 | 54.43 | | 23 | 4-COMe | 2.30 | 2.21 | 1.374 | 12.65 | 54.59 | | 24 | 3,4-diMe | 5.25 | 5.47 | 1.404 | 12.15 | 53.91 | | 25 | 4-SMe | 4.40 | 3.70 | 1.392 | 12.22 | 54.62 | | 26 | 4-tBut | 4.95 | 5.03 | 1.403 | 12.10 | 54.27 | | 27 | 3-COOMe | 3.55 | 3.94 | 1.395 | 12.16 | 54.65 | | 28 | 4-COOMe | 2.44 | 2.28 | 1.377 | 12.55 | 54.77 | | 29 | 3-NO2,4-Me | 2.96 | 3.52 | 1.392 | 12.14 | 54.58 | | 30 | 2-NH2,4-COOH | 5.13 | 4.35 | 1.397 | 12.13 | 53.86 | | 31 | 2-F,3-NO2 | -0.30 | -0.17 | 1.347 | 13.48 | 56.30 | | 32 | 3-CF3 | 3.49 | 3.41 | 1.392 | 12.18 | 55.09 | | 33 | 4-CF3 | 2.58 | 2.55 | 1.383 | 12.35 | 55.14 | | 34 | 4-COOEt | 2.53 | 2.36 | 1.378 | 12.53 | 54.77 | | 35 | 3-SiMe3 | 4.64 | 5.07 | 1.401 | 12.19 | 53.81 | | 36 | 4-SiMe3 | 4.36 | 4.69 | 1.397 | 12.26 | 53.81 | | 37 | 3-SO2Me | 2.69 | 3.11 | 1.390 | 12.22 | 55.33 | | 38 | 3-Br | 3.44 | 3.53 | 1.392 | 12.26 | 55.54 | | 39 | 4-Br | 3.84 | 3.69 | 1.394 | 12.21 | 55.55 | | 40 | 3-SO2NH2 | 2.87 | 3.05 | 1.389 | 12.23 | 55.38 | | 41 | 4-SO2NH2 | 2.08 | 1.69 | 1.376 | 12.44 | 55.31 | | 42 | 4-COOnPr | 2.49 | 2.31 | 1.377 | 12.53 | 54.78 | | 43 | 4-Ph-NH2 | 4.38 | 4.81 | 1.399 | 12.24 | 54.27 | | 44 | 4-CONHNH2 | 2.81 | 2.88 | 1.384 | 12.39 | 54.69 | | 45 | 4-SH | 4.40 | 4.66 | 1.401 | 12.15 | 54.86 | | 46 | 4-SO2Me | 1.48 | 1.85 | 1.377 | 12.43 | 55.28 | | 47 | 4-NHPhOMe | 5.90 | 5.33 | 1.405 | 12.16 | 54.59 | | 48 | 4-Ph | 4.38 | 4.37 | 1.396 | 12.26 | 54.36 | | 49 | 4-NHCOMe | 4.20 | 4.73 | 1.401 | 12.15 | 54.72 | | 50 | 3-COOMe | 5.82 | 3.94 | 1.395 | 12.16 | 54.65 | | 51 | 4-benzoimidazol | 3.65 | 3.30 | 1.386 | 12.40 | 54.60 | |
| --- | --- | --- | --- | --- | --- | --- | --- | --- | --- | --- | --- | --- | --- | --- | --- | --- | --- | --- | --- | --- | --- | --- | --- | --- | --- | --- | --- | --- | --- | --- | --- | --- | --- | --- | --- | --- | --- | --- | --- | --- | --- | --- | --- | --- | --- | --- | --- | --- | --- | --- | --- | --- | --- | --- | --- | --- | --- | --- | --- | --- | --- | --- | --- | --- | --- | --- | --- | --- | --- | --- | --- | --- | --- | --- | --- | --- | --- | --- | --- | --- | --- | --- | --- | --- | --- | --- | --- | --- | --- | --- | --- | --- | --- | --- | --- | --- | --- | --- | --- | --- | --- | --- | --- | --- | --- | --- | --- | --- | --- | --- | --- | --- | --- | --- | --- | --- | --- | --- | --- | --- | --- | --- | --- | --- | --- | --- | --- | --- | --- | --- | --- | --- | --- | --- | --- | --- | --- | --- | --- | --- | --- | --- | --- | --- | --- | --- | --- | --- | --- | --- | --- | --- | --- | --- | --- | --- | --- | --- | --- | --- | --- | --- | --- | --- | --- | --- | --- | --- | --- | --- | --- | --- | --- | --- | --- | --- | --- | --- | --- | --- | --- | --- | --- | --- | --- | --- | --- | --- | --- | --- | --- | --- | --- | --- | --- | --- | --- | --- | --- | --- | --- | --- | --- | --- | --- | --- | --- | --- | --- | --- | --- | --- | --- | --- | --- | --- | --- | --- | --- | --- | --- | --- | --- | --- | --- | --- | --- | --- | --- | --- | --- | --- | --- | --- | --- | --- | --- | --- | --- | --- | --- | --- | --- | --- | --- | --- | --- | --- | --- | --- | --- | --- | --- | --- | --- | --- | --- | --- | --- | --- | --- | --- | --- | --- | --- | --- | --- | --- | --- | --- | --- | --- | --- | --- | --- | --- | --- | --- | --- | --- | --- | --- | --- | --- | --- | --- | --- | --- | --- | --- | --- | --- | --- | --- | --- | --- | --- | --- | --- | --- | --- | --- | --- | --- | --- | --- | --- | --- | --- | --- | --- | --- | --- | --- | --- | --- | --- | --- | --- | --- | --- | --- | --- | --- | --- | --- | --- | --- | --- | --- | --- | --- | --- | --- | --- | --- | --- | --- | --- | --- | --- | --- | --- | --- | --- | --- | --- | --- | --- | --- | --- | --- | --- | --- | --- | --- | --- | --- | --- | --- | --- | --- | --- | --- | --- |

**S.III. Cif files generated after optimization of polymorphic forms of SNM crystals in Material Studio 6.1**

**III.1 α**-SNM

| data_SULAMD08  _audit_creation_date 2013-06-24  _audit_creation_method 'Materials Studio'  _symmetry_space_group_name_H-M 'PBCA'  _symmetry_Int_Tables_number 61  _symmetry_cell_setting orthorhombic  loop_  _symmetry_equiv_pos_as_xyz  x,y,z  -x+1/2,-y,z+1/2  -x,y+1/2,-z+1/2  x+1/2,-y+1/2,-z  -x,-y,-z  x+1/2,y,-z+1/2  x,-y+1/2,z+1/2  -x+1/2,y+1/2,z  _cell_length_a 14.6000  _cell_length_b 5.5720  _cell_length_c 18.4830  _cell_angle_alpha 90.0000  _cell_angle_beta 90.0000  _cell_angle_gamma 90.0000  loop_  _atom_site_label  _atom_site_type_symbol  _atom_site_fract_x  _atom_site_fract_y  _atom_site_fract_z  _atom_site_U_iso_or_equiv  _atom_site_adp_type  _atom_site_occupancy  S1 S 0.89620 0.22875 0.56156 0.00000 Uiso 1.00  O1 O 0.84655 0.00151 0.54796 0.00000 Uiso 1.00  O2 O 0.99463 0.24683 0.54414 0.00000 Uiso 1.00  N1 N 0.86846 0.44040 0.87504 0.00000 Uiso 1.00  N2 N 0.84694 0.43397 0.50861 0.00000 Uiso 1.00  C1 C 0.87351 0.39381 0.80248 0.00000 Uiso 1.00  C2 C 0.83103 0.18746 0.77307 0.00000 Uiso 1.00  C3 C 0.83460 0.14085 0.69935 0.00000 Uiso 1.00  C4 C 0.88088 0.30073 0.65338 0.00000 Uiso 1.00  C5 C 0.92222 0.50922 0.68122 0.00000 Uiso 1.00  C6 C 0.91815 0.55571 0.75483 0.00000 Uiso 1.00  H1 H 0.85055 0.30346 0.90893 0.00000 Uiso 1.00  H2 H 0.91334 0.56053 0.89706 0.00000 Uiso 1.00  H3 H 0.78011 0.46715 0.52393 0.00000 Uiso 1.00  H4 H 0.88603 0.58731 0.50712 0.00000 Uiso 1.00  H5 H 0.79521 0.06357 0.80897 0.00000 Uiso 1.00  H6 H 0.80253 -0.02034 0.67766 0.00000 Uiso 1.00  H7 H 0.95846 0.63342 0.64563 0.00000 Uiso 1.00  H8 H 0.95030 0.71707 0.77645 0.00000 Uiso 1.00  loop_  _geom_bond_atom_site_label_1  _geom_bond_atom_site_label_2  _geom_bond_distance  _geom_bond_site_symmetry_2  _ccdc_geom_bond_type  S1 O1 1.480 . D  S1 O2 1.476 . D  S1 N2 1.668 . S  S1 C4 1.758 . S  N1 C1 1.368 . S  N1 H1 1.021 . S  N1 H2 1.021 . S  N2 H3 1.033 . S  N2 H4 1.028 . S  C1 C2 1.415 . A  C1 C6 1.419 . A  C2 C3 1.388 . A  C2 H5 1.091 . S  C3 C4 1.404 . A  C3 H6 1.089 . S  C4 C5 1.407 . A  C5 H7 1.092 . S  C5 C6 1.386 . A  C6 H8 1.090 . S |
| --- |

**III.2 β**-SNM

| data_SULAMD07  _audit_creation_date 2013-06-24  _audit_creation_method 'Materials Studio'  _symmetry_space_group_name_H-M 'P21/C'  _symmetry_Int_Tables_number 14  _symmetry_cell_setting monoclinic  loop_  _symmetry_equiv_pos_as_xyz  x,y,z  -x,y+1/2,-z+1/2  -x,-y,-z  x,-y+1/2,z+1/2  _cell_length_a 8.8690  _cell_length_b 8.9150  _cell_length_c 9.9620  _cell_angle_alpha 90.0000  _cell_angle_beta 110.4000  _cell_angle_gamma 90.0000  loop_  _atom_site_label  _atom_site_type_symbol  _atom_site_fract_x  _atom_site_fract_y  _atom_site_fract_z  _atom_site_U_iso_or_equiv  _atom_site_adp_type  _atom_site_occupancy  S1 S 0.08228 0.13888 0.78665 0.00000 Uiso 1.00  O1 O 0.00663 0.13367 0.62827 0.00000 Uiso 1.00  O2 O 0.11919 -0.00278 0.86868 0.00000 Uiso 1.00  C1 C 0.26320 0.24024 0.82709 0.00000 Uiso 1.00  C2 C 0.26665 0.37100 0.74894 0.00000 Uiso 1.00  C3 C 0.40309 0.19087 0.93649 0.00000 Uiso 1.00  C4 C 0.54618 0.26940 0.96541 0.00000 Uiso 1.00  C5 C 0.40906 0.45024 0.77898 0.00000 Uiso 1.00  N1 N 0.69334 0.47507 0.91156 0.00000 Uiso 1.00  C6 C 0.55241 0.40038 0.88659 0.00000 Uiso 1.00  N2 N -0.05054 0.22898 0.83959 0.00000 Uiso 1.00  H1 H 0.65528 0.23033 1.04913 0.00000 Uiso 1.00  H2 H 0.15794 0.41177 0.66605 0.00000 Uiso 1.00  H3 H 0.39874 0.09066 0.99757 0.00000 Uiso 1.00  H4 H 0.41097 0.55271 0.71960 0.00000 Uiso 1.00  H5 H 0.79057 0.45062 1.00012 0.00000 Uiso 1.00  H6 H -0.01059 0.24710 0.94827 0.00000 Uiso 1.00  H7 H -0.08578 0.32701 0.78271 0.00000 Uiso 1.00  H8 H 0.69097 0.57860 0.86828 0.00000 Uiso 1.00  loop_  _geom_bond_atom_site_label_1  _geom_bond_atom_site_label_2  _geom_bond_distance  _geom_bond_site_symmetry_2  _ccdc_geom_bond_type  S1 O1 1.485 . D  S1 O2 1.478 . D  S1 C1 1.761 . S  S1 N2 1.656 . S  C1 C2 1.408 . A  C1 C3 1.406 . A  C2 C5 1.386 . A  C2 H2 1.090 . S  C3 C4 1.389 . A  C3 H3 1.089 . S  C4 C6 1.419 . A  C4 H1 1.091 . S  C5 H4 1.092 . S  C5 C6 1.419 . A  N1 C6 1.360 . S  N1 H5 1.019 . S  N1 H8 1.016 . S  N2 H6 1.028 . S  N2 H7 1.029 . S |
| --- |

**III.3 γ**-SNM

| data_SULAMD09  _audit_creation_date 2013-06-24  _audit_creation_method 'Materials Studio'  _symmetry_space_group_name_H-M 'P21/C'  _symmetry_Int_Tables_number 14  _symmetry_cell_setting monoclinic  loop_  _symmetry_equiv_pos_as_xyz  x,y,z  -x,y+1/2,-z+1/2  -x,-y,-z  x,-y+1/2,z+1/2  _cell_length_a 7.8240  _cell_length_b 12.9910  _cell_length_c 7.6200  _cell_angle_alpha 90.0000  _cell_angle_beta 104.9700  _cell_angle_gamma 90.0000  loop_  _atom_site_label  _atom_site_type_symbol  _atom_site_fract_x  _atom_site_fract_y  _atom_site_fract_z  _atom_site_U_iso_or_equiv  _atom_site_adp_type  _atom_site_occupancy  S1 S 0.60201 0.83718 0.04047 0.00000 Uiso 1.00  O1 O 0.59669 0.72398 0.02089 0.00000 Uiso 1.00  O2 O 0.56126 0.90307 -0.12372 0.00000 Uiso 1.00  N1 N 1.31477 0.95357 0.49127 0.00000 Uiso 1.00  N2 N 0.44740 0.86575 0.14755 0.00000 Uiso 1.00  C1 C 1.14781 0.92659 0.39310 0.00000 Uiso 1.00  C2 C 1.08053 0.82625 0.40335 0.00000 Uiso 1.00  C3 C 0.91420 0.79958 0.29638 0.00000 Uiso 1.00  C4 C 0.81338 0.87140 0.17477 0.00000 Uiso 1.00  C5 C 0.87715 0.97181 0.16531 0.00000 Uiso 1.00  C6 C 1.04209 0.99864 0.27371 0.00000 Uiso 1.00  H1 H 1.34165 1.03011 0.50772 0.00000 Uiso 1.00  H2 H 1.37601 0.90904 0.59927 0.00000 Uiso 1.00  H3 H 0.43871 0.94442 0.15930 0.00000 Uiso 1.00  H4 H 0.47461 0.83048 0.27301 0.00000 Uiso 1.00  H5 H 1.15961 0.76986 0.49617 0.00000 Uiso 1.00  H6 H 0.86101 0.72311 0.30679 0.00000 Uiso 1.00  H7 H 0.79903 1.02842 0.07307 0.00000 Uiso 1.00  H8 H 1.09262 1.07632 0.26820 0.00000 Uiso 1.00  loop_  _geom_bond_atom_site_label_1  _geom_bond_atom_site_label_2  _geom_bond_distance  _geom_bond_site_symmetry_2  _ccdc_geom_bond_type  S1 O1 1.478 . D  S1 O2 1.481 . D  S1 N2 1.666 . S  S1 C4 1.762 . S  N1 C1 1.373 . S  N1 H1 1.017 . S  N1 H2 1.019 . S  N2 H3 1.030 . S  N2 H4 1.032 . S  C1 C2 1.416 . A  C1 C6 1.414 . A  C2 C3 1.389 . A  C2 H5 1.093 . S  C3 C4 1.405 . A  C3 H6 1.088 . S  C4 C5 1.405 . A  C5 H7 1.089 . S  C5 C6 1.385 . A  C6 H8 1.089 . S |
| --- |

**III.4 **-SNM

| data_WOBHUR  _audit_creation_date 2013-06-24  _audit_creation_method 'Materials Studio'  _symmetry_space_group_name_H-M 'PBCA'  _symmetry_Int_Tables_number 61  _symmetry_cell_setting orthorhombic  loop_  _symmetry_equiv_pos_as_xyz  x,y,z  -x+1/2,-y,z+1/2  -x,y+1/2,-z+1/2  x+1/2,-y+1/2,-z  -x,-y,-z  x+1/2,y,-z+1/2  x,-y+1/2,z+1/2  -x+1/2,y+1/2,z  _cell_length_a 9.7056  _cell_length_b 8.6794  _cell_length_c 17.8900  _cell_angle_alpha 90.0000  _cell_angle_beta 90.0000  _cell_angle_gamma 90.0000  loop_  _atom_site_label  _atom_site_type_symbol  _atom_site_fract_x  _atom_site_fract_y  _atom_site_fract_z  _atom_site_U_iso_or_equiv  _atom_site_adp_type  _atom_site_occupancy  S1 S 0.42459 0.23116 0.54573 0.00000 Uiso 1.00  O1 O 0.56551 0.18159 0.52455 0.00000 Uiso 1.00  O2 O 0.38597 0.39512 0.54005 0.00000 Uiso 1.00  N1 N 0.35772 0.07500 0.86673 0.00000 Uiso 1.00  H1 H 0.31489 0.15914 0.89902 0.00000 Uiso 1.00  H2 H 0.43969 0.02296 0.89195 0.00000 Uiso 1.00  N2 N 0.32191 0.13855 0.48661 0.00000 Uiso 1.00  H3 H 0.21993 0.16122 0.49735 0.00000 Uiso 1.00  H4 H 0.34332 0.02240 0.48677 0.00000 Uiso 1.00  C1 C 0.36848 0.10547 0.79161 0.00000 Uiso 1.00  C2 C 0.46419 0.02464 0.74670 0.00000 Uiso 1.00  H5 H 0.53087 -0.06164 0.77223 0.00000 Uiso 1.00  C3 C 0.47884 0.05810 0.67118 0.00000 Uiso 1.00  H6 H 0.55730 -0.00101 0.63825 0.00000 Uiso 1.00  C4 C 0.39625 0.17311 0.63883 0.00000 Uiso 1.00  C5 C 0.29582 0.24968 0.68157 0.00000 Uiso 1.00  H7 H 0.23190 0.33927 0.65653 0.00000 Uiso 1.00  C6 C 0.28185 0.21582 0.75706 0.00000 Uiso 1.00  H8 H 0.20633 0.27809 0.79063 0.00000 Uiso 1.00  loop_  _geom_bond_atom_site_label_1  _geom_bond_atom_site_label_2  _geom_bond_distance  _geom_bond_site_symmetry_2  _ccdc_geom_bond_type  S1 O1 1.483 . D  S1 O2 1.475 . D  S1 N2 1.661 . S  S1 C4 1.762 . S  N1 H1 1.020 . S  N1 H2 1.020 . S  N1 C1 1.374 . S  N2 H3 1.027 . S  N2 H4 1.029 . S  C1 C2 1.414 . A  C1 C6 1.416 . A  C2 H5 1.090 . S  C2 C3 1.389 . A  C3 H6 1.091 . S  C3 C4 1.405 . A  C4 C5 1.406 . A  C5 H7 1.091 . S  C5 C6 1.389 . A  C6 H8 1.091 . S |
| --- |
